# This Is the End: Regulation of Rab7 Nucleotide Binding in Endolysosomal Trafficking and Autophagy

**DOI:** 10.3389/fcell.2018.00129

**Published:** 2018-10-02

**Authors:** Christopher Stroupe

**Affiliations:** Department of Molecular Physiology and Biological Physics, University of Virginia School of Medicine, Charlottesville, VA, United States

**Keywords:** Rab GTPase, Rab7, Ypt7p, guanine nucleotide exchange factor, GEF, GTPase-activating protein, GAP, autophagy

## Abstract

Rab7 – or in yeast, Ypt7p – governs membrane trafficking in the late endocytic and autophagic pathways. Rab7 also regulates mitochondrion-lysosome contacts, the sites of mitochondrial fission. Like all Rab GTPases, Rab7 cycles between an “active” GTP-bound form that binds downstream effectors – e.g., the HOPS and retromer complexes and the dynactin-binding Rab-interacting lysosomal protein (RILP) – and an “inactive” GDP-bound form that cannot bind effectors. Accessory proteins regulate the nucleotide binding state of Rab7: guanine nucleotide exchange factors (GEFs) stimulate exchange of bound GDP for GTP, resulting in Rab7 activation, whereas GTPase activating proteins (GAPs) boost Rab7’s GTP hydrolysis activity, thereby inactivating Rab7. This review will discuss the GEF and GAPs that control Rab7 nucleotide binding, and thus regulate Rab7’s activity in endolysosomal trafficking and autophagy. It will also consider how bacterial pathogens manipulate Rab7 nucleotide binding to support intracellular invasion and immune evasion.

## Introduction

Rab GTPases are essential for eukaryotic intracellular membrane and protein trafficking (Zhen and Stenmark, 2015). Specifically, Rab proteins regulate vesicle transport along microtubules and actin filaments (Seabra and Coudrier, 2004; Horgan and McCaffrey, 2011), membrane tethering and docking (Walworth et al., 1989; Mayer and Wickner, 1997), and SNARE complex formation and membrane fusion (Søgaard et al., 1994). Rabs associate with membranes via hydrophobic isoprenyl groups covalently attached at carboxy-terminal cysteines (Calero et al., 2003). Rab GTPases then act by binding to downstream effector proteins and protein complexes (Grosshans et al., 2006).

**FIGURE 1:**
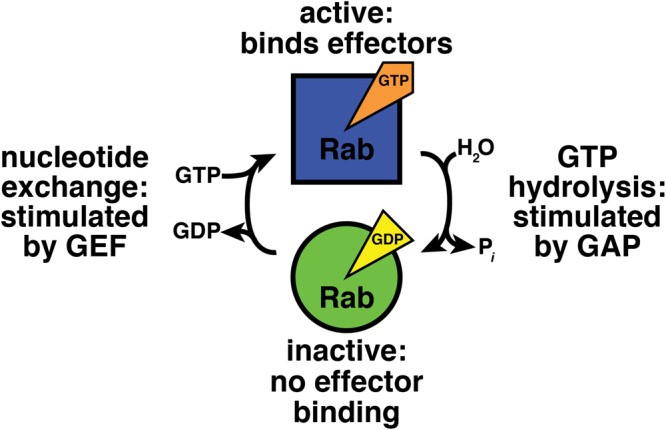
GTPase cycle of nucleotide hydrolysis and exchange. GEF, guanine nucleotide exchange factor; GAP, GTPase activating protein.

Nucleotide binding regulates Rab-effector interactions (**Figure 1**; Walworth et al., 1989; Grosshans et al., 2006). Effectors bind specifically to GTP-bound Rab proteins, and therefore the GTP-bound form can be considered the “active” state (Grosshans et al., 2006). GDP-bound Rabs do not bind effectors and can be regarded as “inactive” (Grosshans et al., 2006). Thus, to understand how Rab GTPases function in their cellular context, we must understand how the cell modulates Rab-nucleotide binding.

Accessory proteins control Rab GTPase nucleotide binding (**Figure 1**; Barr and Lambright, 2010). Guanine nucleotide exchange factors (GEFs) activate Rabs by stimulating exchange of bound GDP for GTP (Ishida et al., 2016; Muller and Goody, 2018). GTPase activating proteins (GAPs) inactivate Rab proteins by boosting their activity for GTP hydrolysis (Fukuda, 2011).

This review will discuss how nucleotide binding by Rab7 and its yeast homolog, Ypt7p, is regulated. Rab7 and Ypt7p govern membrane trafficking in the late endocytic and autophagic pathways (Wichmann et al., 1992; Haas et al., 1995; Kim et al., 1999; Guerra and Bucci, 2016). The specific processes controlled by Rab7 and Ypt7p are diverse, as reflected by the large and diverse set of Rab7/Ypt7p effectors.

The retromer complex is a Rab7 effector that regulates protein sorting in the endocytic pathway (Rojas et al., 2008; Seaman et al., 2009). Retromer directs cargo transport from endosomes to the *trans-*Golgi network (TGN) and plasma membrane (Liu, 2016). Specifically, retromer mediates retrieval of cargo receptors needed for lysosomal enzyme localization, for example the cation-independent mannose 6-phosphate receptor (CI-MPR), to the TGN (Arighi et al., 2004). Retromer also mediates trafficking of endocytosed proteins, e.g., the β2-adrenergic receptor (β2AR) and the type II TGF-β receptor (TβRII), from early endosomes to the plasma membrane (Temkin et al., 2011; Yin et al., 2013). Retromer binds the Rab7 GAP TBC1D5 (Seaman et al., 2009), and this interaction will be discussed in more detail below.

Rab7 also regulates organelle positioning via its effectors Rab7-interacting lysosomal protein (RILP) (Cantalupo et al., 2001; Jordens et al., 2001) and FYVE and coiled-coil containing protein (FYCO1) (Pankiv et al., 2010). RILP recruits the dynein-dynactin motor complex to membranes by binding the p150^Glued^ protein, a subunit of dynactin (Jordens et al., 2001; Johansson et al., 2007). Rab7 thereby promotes minus end-directed transport of late endosomes and lysosomes on microtubules, that is, transport away from the cell periphery and toward the nucleus (Jordens et al., 2001; Johansson et al., 2007). FYCO1 binds the autophagosomal proteins LC3A and LC3B (Olsvik et al., 2015) and is required for plus end-directed transport, i.e., toward the cell periphery, of autophagosomes on microtubules (Pankiv et al., 2010). FYCO1 also is found on late endosomes and lysosomes, and regulates plus end-directed transport of these organelles as well (Pankiv et al., 2010). The precise mechanism by which FYCO1 regulates organelle positioning remains unknown, but it has been proposed to interact directly or indirectly with kinesin (Pankiv et al., 2010).

Rab7 effectors also regulate protein and lipid kinases that are important for autophagy and for endolysosomal trafficking. Ivy1p, the yeast homolog of the metazoan missing in metastasis (MIM) protein, is a Ypt7p effector (Lazar et al., 2002) that also interacts with the Ragulator complex (Numrich et al., 2015), which in turn regulates the target of rapamycin complex 1 (TORC1) in response to cellular amino acid levels (Jewell et al., 2013). Ivy1p promotes TORC1 activity (Numrich et al., 2015), suggesting that Ivy1p plays a role in signal transduction in response to metabolic state (Jewell et al., 2013). Rubicon, a subunit of the endosomal Vps34/class III phosphatidylinositol 3-kinase (PI3KC3) complex, is also a Rab7 effector (Sun et al., 2010). Rubicon negatively regulates Vps34 PI-3-kinase activity (Zhong et al., 2009) and opposes maturation of both autophagosomes (Matsunaga et al., 2009) and endosomes (Sun et al., 2010).

Finally, Rab7 and Ypt7p mediate membrane tethering and fusion. In yeast, the Ypt7p effector responsible for these activities is the homotypic fusion and protein sorting (HOPS)/vacuole protein sorting class C (Vps class C) complex, usually referred to as HOPS (Seals et al., 2000; Wurmser et al., 2000). HOPS is required for membrane tethering (Stroupe et al., 2006) and fusion of lysosomes/vacuoles with late endosomes and autophagosomes (Rieder and Emr, 1997; Jiang et al., 2014; Takats et al., 2014). In yeast, HOPS also is needed for homotypic vacuole fusion (Seals et al., 2000). There is little evidence that HOPS binds Rab7 in metazoans, where HOPS-membrane recruitment appears to be mediated by Arl8b, an Arf-like GTPase (Garg et al., 2011; Khatter et al., 2015). Instead, human Rab7 directly tethers membranes via formation of a Rab7-Rab7 homodimer (Tamura and Mima, 2014).

The following discussion will cover three topics: first, the GEF that activate Rab7/Ypt7p; second, the GAPs that inactivate Rab7/Ypt7p; and third, bacterial effectors, or virulence factors, that modulate Rab7 nucleotide binding to promote intracellular survival and pathogenesis.

## Rab7 Gef: the Mon1-Ccz1 Complex

**FIGURE 2:**
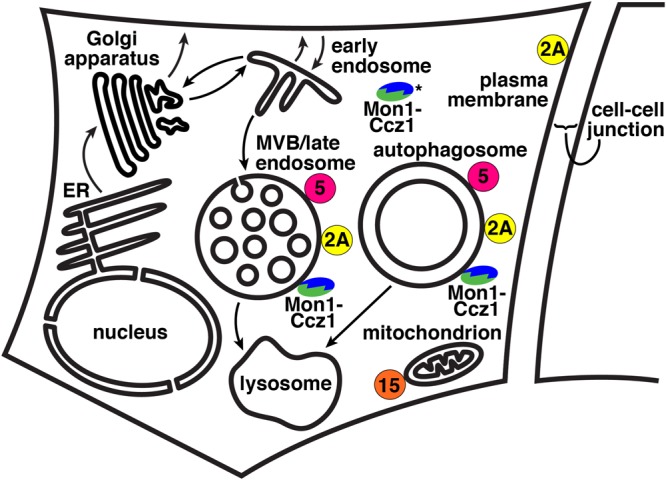
Cellular localizations of Rab GEF and GAPs. Green/blue, Monl-Cczl; yellow, Armus/TBC1D2A; pink, TBC1D5; orange, TBC1D15. MVB, multi-vesicular body; ER, endoplasmic reticulum. Black arrows denote trafficking pathways and represent both vesicular and compartment maturation mechanisms. ^∗^Monl-Cczl has a substantial cytosolic localization, particularly when it is phosphorylated (Lawrence et al., 2014). Not shown, Monl-Cczl also localizes to phagosomes.

The Mon1-Ccz1 complex is the GEF for Rab7 in metazoans and yeast (Kinchen and Ravichandran, 2010; Nordmann et al., 2010; Poteryaev et al., 2010). The yeast HOPS subunit Vps39p has been proposed as a Ypt7p GEF (Wurmser et al., 2000), but purified yeast HOPS has no GEF activity toward Ypt7p (Nordmann et al., 2010). Mon1-Ccz1 is a heterodimer (Wang et al., 2002). Its subunits were identified in yeast via screens for genes whose deletion causes hypersensitivity to the ionophore monensin (Mon1) (Muren et al., 2001) or to high levels of calcium, caffeine, and zinc (Ccz1) (Kucharczyk et al., 1999). Mon1-Ccz1 localizes to late endosomes, autophagosomes, and the cytosol (**Figure 2**; Wang et al., 2002; Poteryaev et al., 2010; Hegedus et al., 2016; Gao et al., 2018). In yeast, Mon1-Ccz1 is present on vacuoles (Lawrence et al., 2014). Mon1-Ccz1 also is found on phagosomes that degrade apoptotic cells (Kinchen and Ravichandran, 2010).

### Biochemical Mechanism

**FIGURE 3:**
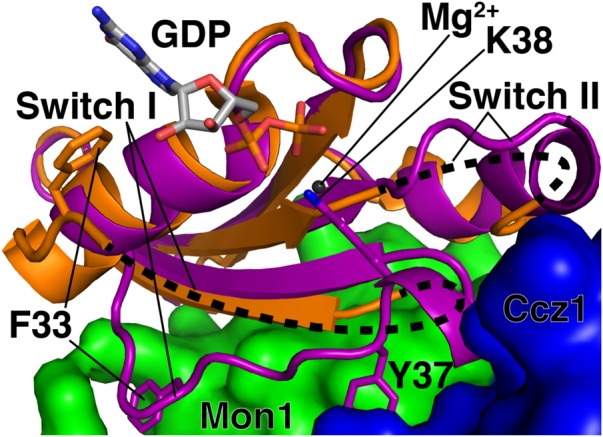
Mechanism of nucleotide exchange catalyzed by the Rab7 GEF Monl-Cczl. Purple, nucleotide-free Rab7; green, Monl; blue, Cczl; orange, Rab7-GDP. The Rab7/Monl-Cczl complex shown here is a homology model, built using Swiss-Model and based on the crystal structure of Chaetomium thermophilum Ypt7 bound to C. thermophilum Monl-Cczl (PDB code 5LDD). The black dashed lines only show the connectivity of the switch I and II regions of Rab7-GDP (PDB code 1KY3). The position of the GDP is also taken from PDB code 1KY3. Figure was made using MacPyMOL.

Mon1-Ccz1 acts by disrupting the nucleotide binding site of Rab7/Ypt7p (Kiontke et al., 2017). **Figure 3** depicts a complex of nucleotide-free Rab7 with Mon1-Ccz1, i.e., a stabilized transition state between GDP- and GTP-bound Rab7 (Kiontke et al., 2017). **Figure 3** shows a homology model of human Rab7/Mon1-Ccz1 (Waterhouse et al., 2018) and is based on the structure of the equivalent proteins from the thermophilic filamentous fungus *Chaetomium theromophilum* (Kiontke et al., 2017).

Mon1-Ccz1 disrupts GDP binding in two ways. First, the “switch I” region of Rab7 (**Figure 3**, purple), which is disordered in Rab7-GDP (**Figure 3**, orange/black dashed lines), binds to a hydrophobic cleft in Mon1-Ccz1 (**Figure 3**, green and blue, respectively). This causes a lysine sidechain from Rab7 to point inward toward the spot where a Mg^2+^ ion is present in Rab7-GDP (**Figure 3**, gray sphere and residue K38; Rak et al., 2004). This lysine presumably disrupts Mg^2+^ binding by charge-charge repulsion. Bound Mg^2+^ is essential for nucleotide binding in small GTPases (Hall and Self, 1986; Burstein and Macara, 1992). Thus, loss of Mg^2^*^+^* is proposed to reduce Rab7’s affinity for GDP (Kiontke et al., 2017). Second, binding of switch I to Mon1-Ccz1 moves a phenylalanine sidechain in Rab7 away from the area where the guanine ring is found in Rab7-GDP (**Figure 3**, residue F33). This phenylalanine makes a stabilizing edge-face aromatic interaction with the guanine in Rab7-GDP (Rak et al., 2004). Thus, loss of this interaction should also reduce Rab7 affinity for GDP.

The mechanism of action of Mon1-Ccz1 is quite different from that of other Rab GEFs (Ishida et al., 2016; Muller and Goody, 2018). Mon1-Ccz1 does not belong to the largest and most well-known class of Rab GEFs, the differentially expressed in normal and neoplastic cells (DENN) domain proteins (Ishida et al., 2016). Rather, the core element in both Mon1 and Ccz1 is a “longin” domain (Cabrera et al., 2014), a small α-β-α sandwich often found in proteins that regulate intracellular traffic (De Franceschi et al., 2014). Longin domains are versatile: some longin domains mediate nucleotide exchange on Rab GTPases, for example as part of the TRAPP complexes (Kim et al., 2006; Cai et al., 2008), whereas others are found in SNARE proteins and appear to have no GEF activity (Rossi et al., 2004).

### Cellular Role of Mon1-Ccz1

**FIGURE 4:**
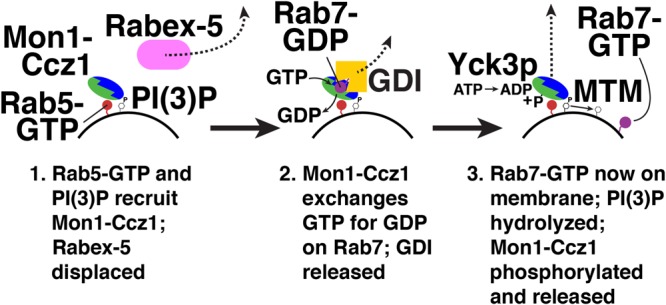
Rab conversion from Rab5 to Rab7. Red, Rab5; blue/green, Monl-Cczl; pink, Rabex-5; purple, Rab7; yellow, GDI. Yck3p, yeast vacuolar casein kinase; MTM, myotubularin PI-3 phosphatase. Not shown, a GDF (GDI displacement factor) may disrupt the Rab7-GDI complex (Dirac-Svejstrup et al., 1997).

Mon1-Ccz1 is not only a Rab7 GEF, but also a Rab5 effector (Kinchen and Ravichandran, 2010). Rab5 binding to Mon1-Ccz1 initiates “Rab conversion” on endosomes (**Figure 4**; Rink et al., 2005). Here, active Rab5 recruits Mon1-Ccz1 to early endosomes (Kinchen and Ravichandran, 2010). Mon1-Ccz1 also binds to PI(3)P (Poteryaev et al., 2010), which is enriched on early endosomes (Gillooly et al., 2000). Mon1-Ccz1 then catalyzes nucleotide exchange on Rab7-GDP, which is extracted from membranes and held in a soluble, cytosolic state by the GDP dissociation inhibitor (GDI) (Araki et al., 1990; Garrett et al., 1994). GDI interacts only with GDP-bound Rab proteins (Araki et al., 1990; Garrett et al., 1994). Thus, nucleotide exchange triggers Rab7-GTP membrane association (Poteryaev et al., 2010). A GDI displacement factor (GDF) may facilitate Mon1-Ccz1 activity by disrupting Rab7-GDI binding (Dirac-Svejstrup et al., 1997). Mon1-Ccz1 also displaces the GEF for Rab5, Rabex-5, from membranes (Poteryaev et al., 2010). This reduces active Rab5 levels near Rab7-GTP. As a result of these recruitment and displacement actions, active Rab7 replaces active Rab5, thereby converting an early endosome to a late endosome. Rab7 continues this maturation process by recruiting its own effectors (Seals et al., 2000; Wurmser et al., 2000; Cantalupo et al., 2001; Jordens et al., 2001; Lazar et al., 2002; Pankiv et al., 2010; Sun et al., 2010).

Recruitment of a Rab5 GAP by Rab7 could, in principle, also promote Rab conversion. Such a feedback loop has been observed in the secretory pathway in yeast. Here, the Golgi-localized Rab, Ypt32p, binds a GAP for an ER-resident Rab, Ypt1p (Rivera-Molina and Novick, 2009). Membrane recruitment of this GAP promotes conversion of Ypt1p-positive membranes to Ypt32p-positive membranes (Rivera-Molina and Novick, 2009). Overexpression of Mon1-Ccz1 or GTPase-deficient Ypt7p inactivates the yeast Rab5 homolog, Vps21p (Rana et al., 2015). This suggests that a “counter-current” (Rivera-Molina and Novick, 2009) of Rab inactivation does operate on late endosomes. However, no interaction between a Rab5/Vps21p GAP and Rab7/Ypt7p, or a Rab7/Ypt7p effector, has yet been found.

Termination of Mon1-Ccz1 action is mediated by post-translational modification. In yeast, the vacuolar casein kinase Yck3p phosphorylates Mon1p (**Figure 4**; Lawrence et al., 2014). Phosphorylated Mon1-Ccz1 then dissociates from vacuoles (Lawrence et al., 2014). Mon1-Ccz1 dissociates from lysosomes in mammalian cells, though it is not yet known how this dissociation is triggered (Yasuda et al., 2016).

Another possible mechanism for Mon1-Ccz1 inactivation is PI(3)P hydrolysis, which would weaken Mon1-Ccz1 membrane binding (**Figure 4**; Poteryaev et al., 2010). Myotubularin phosphatases (MTMs) hydrolyze the 3-phosphate of PI(3)P on endosomes and autophagosomes (Blondeau et al., 2000; Taylor et al., 2000; Velichkova et al., 2010; Wu et al., 2014). In *Drosophila*, MTM1 is required for lysosomal function (Velichkova et al., 2010). In *C. elegans*, MTM-3 is needed for autophagosome-lysosome fusion (Wu et al., 2014). These results suggest that PI(3)P hydrolysis and Mon1-Ccz1 release play a role in endosome maturation and autophagy.

Binding of Mon1-Ccz1 to LC3 proteins also promotes Mon1-Ccz1 function in autophagy. In yeast, Mon1-Ccz1 binds the LC3 protein Atg8p via LIR motifs in Ccz1 (Gao et al., 2018). This binding is needed for Mon1-Ccz1 association with autophagosomes. Mon1-Ccz1/Atg8p binding also activates the GEF activity of Mon1-Ccz1 activity for Ypt7p (Gao et al., 2018). Membrane recruitment may activate Mon1-Ccz1 by increasing its local concentration near membrane-associated Ypt7p, but allosteric activation upon Atg8p binding cannot be ruled out. Mon1-Ccz1 binding to metazoan LC3 proteins has not been observed, but the LIR motifs in Ccz1 are also found in mammals (Gao et al., 2018).

There are few direct connections between Mon1-Ccz1 and human disease, though autophagy is central to many aspects of human health, including cancer and neuronal/cardiac ischemia (Choi et al., 2013). However, Ccz1 is post-transcriptionally downregulated by miR-1, a microRNA that causes cardiac arrhythmias when overexpressed (Yang et al., 2007; Su et al., 2017). This suggests that Mon1-Ccz1-dependent autophagy or mitophagy suppresses arrhythmias, perhaps by removing damaged mitochondria that generate aberrant intracellular ion fluxes in cardiomyocytes (Brown and O’Rourke, 2010).

### Summary

The sole known Rab7/Ypt7p GEF, Mon1-Ccz1, is also a Rab5 effector (Kinchen and Ravichandran, 2010). Mon1-Ccz1 thereby mediates Rab5-dependent recruitment of Rab7, a process termed “Rab conversion” (**Figure 4**; Rink et al., 2005). Binding of Mon1-Ccz1 to LC3 proteins also promotes Mon1-Ccz1 association with autophagosomes (Gao et al., 2018). PI(3)P binding is needed for Mon1-Ccz1 membrane association (Poteryaev et al., 2010). Together, these interactions support Mon1-Ccz1 action in endocytic trafficking and autophagy. Mon1-Ccz1 release from membranes is triggered by its phosphorylation (Lawrence et al., 2014), and possibly by PI(3)P dephosphorylation (Wu et al., 2014), permitting Rab7 deactivation and GDI extraction.

## Rab7 Gaps

### General Biochemical Mechanism of Rab GAP Action

**FIGURE 5:**
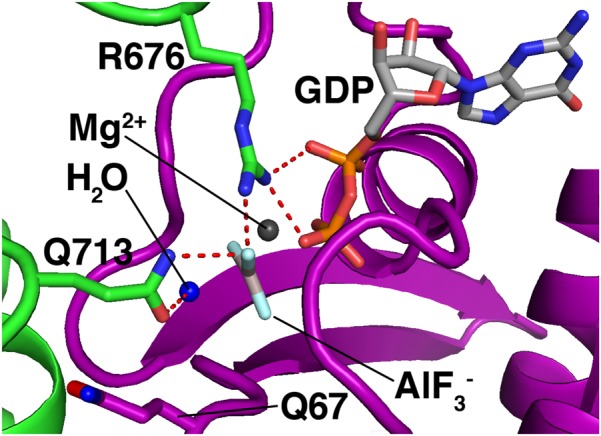
Mechanism of GTP hydrolysis by a Rab GAP. Purple, Rab7; green, Armus/TBC1D2A. The Rab7/Armus complex shown here is a homology model of human Rab7 bound to the TBC domain of human Armus, built using Swiss-Model and based on the crystal structure of Rab33 in complex with Gypl (PDB code 2G77). Figure was made using MacPyMOL.

Rab GAPs use the conserved Tre2/Bub2/Cdc16 (TBC) domain to activate GTP hydrolysis (Fukuda, 2011). **Figure 5** shows the mechanism by which a TBC domain (green) modulates the active site of a Rab GTPase (purple). This figure depicts a homology model (Waterhouse et al., 2018) of Rab7 bound to the TBC domain of the Rab7 GAP Armus/TBC1D2A, and is based on the crystal structure of Rab33 bound to the Gyp1p TBC domain (Pan et al., 2006). A conserved glutamine from the GAP (Q713) activates a water molecule (blue) for hydrolysis. Meanwhile, a conserved arginine from the GAP (R676) provides positive charge to stabilize the GDP leaving group and the planar transition-state form of the γ phosphate, which is mimicked by an AlF_3_^-^ molecule (Pan et al., 2006).

This “dual-finger mechanism” (Pan et al., 2006) differs from the mechanism of Ras GAPs, which use a single “arginine finger” to stabilize the transition state (Scheffzek et al., 1998). This difference is important because in Ras GAP-mediated GTP hydrolysis, the hydrolyzing water is activated by a glutamine from the Ras GTPase itself (Scheffzek et al., 1998). In Rab GTPases, the equivalent glutamine is not needed for GAP-mediated GTP hydrolysis, and is flipped away from the active site in the Rab-GAP complex (**Figure 5**, Q67, bottom left). As a result, Rab GAPs can stimulate GTP hydrolysis by mutant Rab GTPases lacking this glutamine, though the stimulated rate is lower than that of GAP-stimulated wild-type Rab proteins by up to an order of magnitude (De Antoni et al., 2002; Pan et al., 2006).

The conserved glutamine and arginine mentioned above account for most of the GTPase-activating function of Rab GAPs. However, there are at least 27 proteins containing TBC domains in the human genome, and roughly 70 Rab GTPases. There is no satisfactory method for predicting specificity from a TBC domain’s sequence. Rather, researchers have determined TBC domains’ specificity empirically, by testing them for GAP activity against panels of Rab GTPases (Itoh et al., 2006; Pan et al., 2006; Fuchs et al., 2007).

**FIGURE 6:**
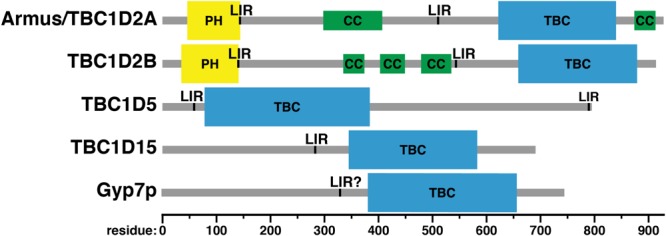
Domain structure of Rab GAPs. PH, Phox homology domain; LIR, LC3-interacting region; CC, predicted coiled-coil; TBC: Tre-2/Bub2/Cdcl6 domain. Domain assignments were made using the Simple Modular Architecture Research Tool (SMART) server, http://smart.embl-heidelberg.de/help/smart_about.shtml.

Accessory domains in Rab GAPs (**Figure 6**) may determine Rab specificity by constraining Rab GAPs’ cellular localization (**Figure 2**). No known Rab GAP consists of only a TBC domain. The domain architecture of Rab GAPs is diverse: they can contain lipid binding domains, coiled-coil motifs, LC3-interacting regions (LIRs), domains that bind to other small GTPases, and other domains with GAP and GEF activity (Fukuda, 2011). Some accessory domains may determine Rab GAP localization via interactions with phospholipids and other proteins (**Table 1**). Thus, proximity of Rab GAPs to particular Rab GTPases, which themselves have distinct cellular localizations (Li and Marlin, 2015), may dictate which Rab GAPs act on which Rabs.

**Table 1:** Rab7/Ypt7p GTPase activating proteins (GAPs).

Name	Cellular location	Other interactions	Effect of overexpression	Effect of knockdown or
				dominant negative mutant
Armus/TBC1D2A/ TBC1D2/TBC-2 (*C. elegans*)	Plasma membrane/ cell-cell junctions (Frasa et al., 2010) Late endosomes (Chotard et al., 2010; Jaber et al., 2016) Autophagosomes (Carroll et al., 2013)	E-cadherin (Frasa et al., 2010) Rac1 (Frasa et al., 2010) LC3A (Popovic et al., 2012; Carroll et al., 2013), GABARAP-L1 (Popovic et al., 2012) Leucine-rich repeat kinase 1 (LRRK1) (Toyofuku et al., 2015) PI(3)P, PI(4)P (Jaber et al., 2016; Law et al., 2017)	Reduction of EGF-induced cell scattering (Frasa et al., 2010)	Autophagosome accumulation, reduction of starvation-induced LC3-II degradation (Carroll et al., 2013; Toyofuku et al., 2015) Endosome accumulation (Chotard et al., 2010) Impaired EGF, LDL, *trans*-ferrin uptake (Serva et al., 2012)
TBC1D2B	n.d.	LC3A (Popovic et al., 2012), LC3B (Behrends et al., 2010), LC3C (Behrends et al., 2010), GABARAP (Behrends et al., 2010), GABARAP-L1 (Behrends et al., 2010; Popovic et al., 2012), GABARAP-L2 (Behrends et al., 2010)	n.d.	n.d.
TBC1D5	Late endosomes (Seaman et al., 2009) Autophagosomes (Popovic et al., 2012)	Retromer (via Vps29) (Seaman et al., 2009) LC3A (Popovic et al., 2012; Roy et al., 2017), LC3C (Roy et al., 2017), GABARAP-L1 (Popovic et al., 2012) Atg9 (Popovic and Dikic, 2014) Ulk1 (Popovic and Dikic, 2014)	Reduced retromer-membrane association (Seaman et al., 2009)	Reduced autophagosome formation (Popovic et al., 2012) Retromer cargo accumulation in endosomes (Jia et al., 2016) Enhanced retromer activity (Seaman et al., 2018)
TBC1D15	Mitochondria (Onoue et al., 2013; Yamano et al., 2014)	Fis1 (Onoue et al., 2013) LC3A (Yamano et al., 2014), LC3B (Behrends et al., 2010; Yamano et al., 2014), LC3C (Behrends et al., 2010; Yamano et al., 2014), GABARAP (Behrends et al., 2010; Yamano et al., 2014), GABARAP-L1 (Behrends et al., 2010; Yamano et al., 2014), GABARAP-L2 (Behrends et al., 2010; Yamano et al., 2014) TBC1D17 (Yamano et al., 2014) Vac14 (Schulze et al., 2014) Rab5 (but has no GAP activity for Rab5) (Itoh et al., 2006)	Increased number of lysosomes per cell (Peralta et al., 2010) Delayed cell death upon growth factor withdrawal (Peralta et al., 2010) GAP-deficient mutants block mitochondrial fission (Wong et al., 2018)	Elongated mitochondria (Onoue et al., 2013) LC3 accumulation during mitophagy (Yamano et al., 2014) Reduced clearance of damaged mitochondria (Yamano et al., 2014) Little effect on starvation-induced autophagy (Yamano et al., 2014)
Gyp7p (yeast)	Cytosol	None identified	Reduced vacuole fusion upon hypo-osmotic shock (Brett et al., 2008)	Small decrease in vacuole fragmentation (Brett et al., 2008)

### Armus/TBC1D2A

The first Rab7-specific GAP to be described, Armus/TBC1D2A, was discovered via a two-hybrid screen for effectors of the Rac1 GTPase (Frasa et al., 2010). In this study, Armus was found to regulate cell-cell adhesion and E-cadherin degradation (Frasa et al., 2010). Overexpression of a construct containing the Armus TBC domain and carboxy-terminal coiled-coil motif (**Figure 6**) – which localizes to the plasma membrane at cell-cell junctions (**Figure 2**) – blocks endocytosis and degradation of the cell adhesion molecule E-cadherin (Frasa et al., 2010). This construct also stabilizes cell-cell contacts in keratinocytes (Frasa et al., 2010). This stabilization may be caused by reduction in active Rab7 and a concomitant reduction in transport to the lysosome via the endocytic pathway.

Counterintuitively, siRNA-mediated knockdown of Armus expression also stabilizes cell-cell junctions (Frasa et al., 2010). Likely this is because GDI delivers Rab7 to maturing endosomes, as described above (**Figure 4**; Poteryaev et al., 2010). GDI binds and extracts only GDP-bound Rab proteins from membranes (Araki et al., 1990; Garrett et al., 1994). Thus, if Rab7 GTP hydrolysis is reduced via Armus knockdown, formation of the soluble Rab7-GDI complex and Rab7 delivery to endosomes also will be reduced. As evidence for the importance of GTP hydrolysis for proper Rab7 localization, knockout of another Rab7 GAP, TBC1D5, redistributes Rab7 from late endosomes to lysosomes (Jimenez-Orgaz et al., 2018). Additionally, a GTPase-deficient mutant of the yeast exocytic Rab Sec4p (Q79L) is a cold-sensitive loss-of-function allele that causes accumulation of secretory vesicles and reduced secretion (Walworth et al., 1992).

Armus also acts in autophagy (Carroll et al., 2013). When Armus is overexpressed, enlarged autophagosomes accumulate, whereas reduction of Armus expression blocks autophagic flux (Carroll et al., 2013). Supporting its role in autophagy, Armus binds the autophagosomal protein LC3 (Carroll et al., 2013). Armus does this via two LC3-interacting regions (LIRs), one just after its amino-terminal pleckstrin homology (PH) domain and the second near its carboxy terminus (**Figure 6**; Carroll et al., 2013). This study examined Armus binding to one of the six human LC3 proteins, LC3A (Carroll et al., 2013), but other work has shown that Armus binds both LC3A and GABARAP-L1 (Popovic et al., 2012). Thus, Armus appears to link the action of Rac1 and Rab7 during autophagy. Rac1-GTP is reduced in starvation-induced autophagy (Carroll et al., 2013). This suggests that decreased Rac1-GTP causes decreased Armus recruitment, which then would boost Rab7-GTP levels and promote autophagosome-lysosome fusion.

How does the Armus PH domain (**Figure 6**) affect Armus function? Knockdown in cultured mammalian cells of Vps34/PI3KC3, the kinase that phosphorylates PI (phosphatidylinositol) to PI(3)P (phosphatidylinositol-3-phosphate) on endosomes, blocks Armus recruitment to membranes (Jaber et al., 2016). Vps34/PI3KC3 knockdown also causes increased Rab7-GTP levels, enlarged late endosomes, and impaired lysosomal cargo delivery (Jaber et al., 2016). The Armus PH domain binds PI(3)P and PI(4)P in lipid overlay and liposome floatation assays (Jaber et al., 2016; Law et al., 2017). Thus, the PH domain of Armus likely helps recruit it to endosomal membranes by binding PI(3)P.

The *C. elegans* Armus homolog, TBC-2, has *in vitro* GAP activity not only for Rab7 (i.e., RAB-7), but also Rab5 (i.e., RAB-5), which governs trafficking to early endosomes (Chotard et al., 2010). However, this does not necessarily conflict with the role of mammalian Armus in inactivating Rab7. First, this study used only the TBC domain of *C. elegans* TBC-2, which, as discussed above, might not contain all of the determinants for Rab specificity (Chotard et al., 2010). Second, even if both mammalian Armus and *C. elegans* TBC-2 have dual specificity for Rab5 and Rab7, this might be an elegant method of inactivating Rab5 on late endosomes, i.e., a proofreading or mop-up function. Finally, Armus and TBC-2 simply may have different functions in their respective organisms.

The Roco kinase leucine-rich repeat kinase 1 (LRRK1) also regulates Armus activity (Toyofuku et al., 2015). Autophagosome-lysosome fusion is impaired in LRRK^-/-^ knockout cells (Toyofuku et al., 2015). Rab7-GTP levels also are elevated, and are further elevated during autophagy (Toyofuku et al., 2015). LRRK1 phosphorylation blocks an intramolecular interaction between the PH and TBC domains of Armus, though neither the binding interface nor the site of phosphorylation has yet been identified (Toyofuku et al., 2015). Thus, phosphorylation of Armus by LRRK1 may relieve an autoinhibitory interaction between its PH and TBC domains, permitting PI(3)P binding, GAP activity, or both. Interaction with another protein, perhaps via the coiled-coil domain of Armus (**Figure 6**), that modulates Armus membrane recruitment and/or activity also cannot be ruled out. LRRK1 is closely related to LRRK2, which is mutated in familial Parkinson’s disease (Zimprich et al., 2004). LRRK2 has not been shown to phosphorylate Armus. Nevertheless, cycling of Rab7 between membrane and cytosol – which requires GTP hydrolysis by Rab7 (Araki et al., 1990; Garrett et al., 1994) – is decreased in cells with Parkinson’s disease-linked LRRK2 mutations (Gomez-Suaga et al., 2014).

Armus has other links to human disease. An Armus variant termed PARIS-1 was found in a screen for antigens recognized by sera from prostate cancer patients (Zhou et al., 2002). PARIS-1 is identical to Armus, except for a deletion of 11 amino acids in its TBC domain (residues 819–829). The biochemical effect of this deletion is not obvious: PARIS-1 is stably expressed (Zhou et al., 2002), and in the crystal structure of the Rab33-Gyp1p TBC domain complex used to make **Figure 5**, these residues do not directly contact the Rab (Pan et al., 2006). Perhaps these residues stabilize structural elements that are important for GAP activity. In another link between Armus and cancer, Armus expression is inhibited by a microRNA (miRNA) from a cluster of six miRNAs, miR-17-92/oncomir-1 (Serva et al., 2012), that is overexpressed or deleted in many cancers (He et al., 2005; Zhang et al., 2006). Finally, a genome-wide association study of multiple sclerosis (MS) patients found an association with a single nucleotide polymorphism in an intron from the gene encoding Armus (Baranzini et al., 2009). This finding has been replicated in a follow-up study of a different group of MS patients (Schmied et al., 2012). However, the basis for this linkage remains unknown.

### TBC1D2B

This protein is not a splice variant of Armus, i.e., TBC1D2 or TBC1D2A (**Table 1**), but rather is encoded by a different gene, with 63% protein sequence identity and a virtually identical domain structure (**Figure 6**; Letunic et al., 2015; Sievers and Higgins, 2018). However, the Rab specificity of TBC1D2B is unknown. It has been included in this review because of its similarity to Armus.

TBC1D2B also binds LC3 proteins. A proteomic study of the six human LC3 proteins found that TBC1D2B associates with five: LC3B, LC3C, GABARAP, GABARAP-L1, and GABARAP-L2 (**Table 1**; Behrends et al., 2010). A low-throughput study of TBC domain protein association showed that TBC1D2B also associates with LC3A (**Table 1**; Popovic et al., 2012). How TBC1D2B binds LC3 proteins is unknown, but the LIRs in Armus (residues 142–146 and 542–546; Carroll et al., 2013) are also present in TBC1D2B (**Figure 6**).

### TBC1D5

TBC1D5 was first identified as a retromer-binding protein (Seaman et al., 2009). As described in the Introduction, retromer regulates protein sorting in the endocytic pathway (Liu, 2016). Retromer is needed for endosome-to-TGN retrieval of CI-MPR, which ferries hydrolytic enzymes to lysosomes (Arighi et al., 2004). Retromer also mediates recycling of cell-surface receptors, e.g., β2AR and TβRII, from endosomes to the plasma membrane (Temkin et al., 2011; Yin et al., 2013).

Retromer is a five-subunit complex consisting of two subcomplexes: a cargo-binding trimer of Vps26, Vps29, and Vps35, and a membrane-binding dimer of sorting nexins (Liu, 2016). The exact set of sorting nexins that bind Vps26/29/35 is variable (Liu, 2016). Differences in sorting nexin composition affect cargo selection and the trafficking pathway in which any particular retromer complex acts (Liu, 2016). Retromer is a Rab7 and Ypt7p effector and is recruited to membranes by binding to Rab7/Ypt7p (Rojas et al., 2008; Seaman et al., 2009) and through direct membrane binding by phox homology (PX) and bin/amphiphysin/Rvs (BAR) domains in the sorting nexins (Xu et al., 2001; Carlton et al., 2004).

TBC1D5 binds retromer via Vps29 (Jia et al., 2016). This interaction may promote retromer function: shRNA-mediated knockdown of TBC1D5 expression reduces CI-MPR retrieval to the TGN (Popovic et al., 2012). TBC1D5 knockdown also may suppress retromer function indirectly, by affecting Rab7 activation and/or localization: CRISPR-mediated TBC1D5 knockout markedly increases Rab7 co-localization with the lysosomal marker LAMP2 (Jimenez-Orgaz et al., 2018).

It should be mentioned that the TBC1D5’s effect on retromer activity is not yet settled: a recent report suggests that TBC1D5 knockdown in fact boosts retromer activity (Seaman et al., 2018). This discrepancy may be due to the degree of TBC1D5 knockdown. Complete TBC1D5 knockout forces Rab7 onto lysosomes, where it cannot bind and activate the retromer (Jimenez-Orgaz et al., 2018). In contrast, incomplete TBC1D5 knockdown may allow enough Rab7 on late endosomes/MVB’s to support retromer activity – and the reduction in TBC1D5 expression may in fact boost the amount of active Rab7.

Like Armus, TBC1D5 co-localizes with LC3 and plays a role in autophagy (Popovic et al., 2012). TBC1D5 also is needed for mitophagy (Jimenez-Orgaz et al., 2018), a specialized form of autophagy that delivers mitochondria to lysosomes for destruction (Kanki and Klionsky, 2010). TBC1D5 contains two LIR motifs (**Figure 6**), and both are required for co-localization with LC3 (Popovic et al., 2012). However, the specific role of TBC1D5 in autophagy differs from that of Armus. Knockdown of TBC1D5 expression by shRNAs prevents accumulation of punctate LC3-positive structures, i.e., autophagosomes (Popovic et al., 2012). Bafilomycin treatment, which blocks autophagosome-lysosome fusion (Yamamoto et al., 1998), does not cause autophagosome buildup in TBC1D5-depleted cells (Popovic et al., 2012). Thus, the defect in TBC1D5-depleted cells lies in autophagosome formation, not consumption. In agreement with this finding, TBC1D5 also interacts with Atg9 and Ulk1, an integral membrane protein and a kinase, respectively, that initiate autophagosome biogenesis (Young et al., 2006; Zavodszky et al., 2013; Popovic and Dikic, 2014).

How do these dual binding specificities, to LC3 proteins and the retromer, relate to one another? Experiments using purified proteins show that LC3A competes with the retromer subunit Vps29 for TBC1D5 binding (Popovic et al., 2012). In agreement with this result, TBC1D5 co-localizes with Vps35 and LC3B, but Vps35 and LC3B do not themselves co-localize (Popovic et al., 2012). Accordingly, the amino-terminal LIR motif in TBC1D5 is needed for retromer-TBC1D5 binding (Popovic et al., 2012). Autophagy triggers the switch between these alternate binding modes: upon starvation, TBC1D5-LC3A co-localization is sharply increased, whereas TBC1D5-retromer binding becomes nearly undetectable (Roy et al., 2017). This effect is autophagy-dependent and not merely triggered by starvation, which does not block TBC1D5-retromer binding in cells lacking the autophagy regulator Atg7 (Roy et al., 2017). Retromer-mediated recycling of the GLUT1 glucose transporter from endosomes to the plasma membrane is increased during starvation (Roy et al., 2017). This effect might be caused by TBC1D5-LC3A binding during autophagy (Roy et al., 2017), which sequesters TBC1D5 and permits Rab7 activation in the vicinity of the retromer.

### TBC1D15

This Rab7 GAP is localized to mitochondria (**Figure 2**) and is involved primarily in mitochondrial physiology (Onoue et al., 2013). TBC1D15 binds the mitochondrial fission regulator Fis1, and TBC1D15’s mitochondrial localization is dependent on Fis1 (Onoue et al., 2013). When TBC1D15 or Fis1 is knocked out, autophagosomes accumulate during valinomycin-induced mitophagy (Yamano et al., 2014). Correspondingly, mitophagy is reduced when TBC1D15 or Fis1 is knocked out (Yamano et al., 2014). In contrast, TBC1D15 knockout has little effect on starvation-induced autophagy (Yamano et al., 2014). TBC1D15 differs from TBC1D5 in this respect: TBC1D5 is needed both for mitophagy (Jimenez-Orgaz et al., 2018) and bulk autophagy (Popovic et al., 2012). TBC1D15 also interacts with LC3 proteins, as shown both by pulldown with GST-tagged LC3 proteins and using proteomic methods (Behrends et al., 2010; Yamano et al., 2014). A LIR motif responsible for this binding was found in TBC1D15 using mutagenesis (**Figure 6**; Yamano et al., 2014).

These results have led to a model in which TBC1D15’s interactions with Fis1 and LC3 proteins hold it in proximity to both mitochondria and autophagosomes (Yamano et al., 2014). In this model, TBC1D15 supports formation of autophagosomes around damaged mitochondria (Yamano et al., 2014). In the absence of TBC1D15, an excess of Rab7-GTP in apposition to mitochondria causes autophagosomes to grow larger than usual. The mechanism by which Rab7-GTP promotes autophagosome expansion is unknown. However, Rab7 knockdown in TBC1D15^-/-^ cells suppresses autophagosome expansion around mitochondria, suggesting that Rab7 indeed plays a role in this process (Yamano et al., 2014).

TBC1D15 also regulates mitochondrion-lysosome contacts that are not involved in mitophagy (Wong et al., 2018). These contacts are dynamic, and TBC1D15-stimulated Rab7 GTP hydrolysis is required for their termination (Wong et al., 2018). Mitochondrial fission occurs at these lysosomal contact sites (Wong et al., 2018). Expression of GTPase-deficient Rab7 mutants or GAP-deficient TBC1D15 mutants sharply reduces mitochondrial fission at lysosomal contacts and causes mitochondrial elongation (Wong et al., 2018). Thus, TBC1D15-stimulated Rab7 GTP hydrolysis regulates mitochondrial morphology even when mitophagy is not induced (Wong et al., 2018). Expression of a Fis1 mutant that cannot bind TBC1D15 also blocks mitochondrial fission at lysosomal contacts, suggesting that Fis1 mediates TBC1D15 recruitment even in the absence of mitophagy (Wong et al., 2018).

Additional interactions link TBC1D15 to other aspects of membrane trafficking. Proteomic approaches identified an interaction between TBC1D15 and Vac14, a scaffolding protein that stimulates PIKfyve, the kinase that generates PI(3,5)P_2_ from PI(3)P (Schulze et al., 2014). PI(3,5)P_2_ is essential for proper lysosomal pH and ion homeostasis (Hasegawa et al., 2017). The significance of the TBC1D15-Vac14 interaction is unknown, but may be a mechanism by which PI(3,5)P_2_ generation is coordinated with Rab7 inactivation on lysosomes. TBC1D15 also interacts with the early endosomal Rab GTPase Rab5 (Itoh et al., 2006). However, TBCD15 does not appear to have GAP activity toward Rab5 (Itoh et al., 2006). Perhaps Rab5-TBC1D15 binding recruits a small amount of TBC1D15 to early endosomes, preventing Rab7 activation there. Finally, TBC1D15 interacts with the Rab GAP TBC1D17 (Yamano et al., 2014), though neither the target of TBC1D17 nor the role of the TBC1D15-TBC1D17 interaction has been determined.

### Gyp7p

Gyp7p is the GAP for Ypt7p, the yeast vacuolar Rab GTPase (Vollmer et al., 1999; Brett et al., 2008). Deletion of the gene encoding Gyp7p has little effect on cell growth or vacuole morphology, other than a small increase in vacuole size (Brett et al., 2008). This is probably due to increased homotypic vacuole-vacuole fusion, a process that requires Ypt7p (Haas et al., 1995). Gyp7p overexpression does, however, cause vacuole fragmentation (Brett et al., 2008). Gyp7p overexpression also blocks vacuole fusion upon hypo-osmotic shock (Brett et al., 2008). Deletion of the gene for Gyp7p does not affect autophagy, though autophagy is known to require Ypt7p (Wurmser et al., 2000).

Little is known about the domain structure of Gyp7p, other than the presence of a TBC domain (**Figure 6**). Overall, the positioning of Gyp7p’s TBC domain is similar to that of TBC1D15, toward the carboxy terminus of the protein (**Figure 6**). No conserved domains have been identified in the amino-terminal regions of either TBC1D15 or Gyp7p, except for TBC1D15’s LIR motif (Yamano et al., 2014). This motif may be conserved in Gyp7p: the sequence of TBC1D15’s LIR, from residues 280–283, is FEVI, whereas Gyp7p contains a sequence of FNDL in roughly the same region, from residues 329–332. The core LIR consensus sequence is [WFY]xx[ILV], though neighboring residues can affect LC3 family protein binding (Jacomin et al., 2016). However, Gyp7p has no known role in autophagy and is not known to bind Atg8p, the yeast LC3 protein, nor is there any known effect of mutating these residues.

### Summary

The Rab7 GAPs have diverse cellular roles (**Table 1**). Armus/TBC1D2A is needed for endocytosis of cell adhesion molecules and modulation of cell-cell contacts (Frasa et al., 2010). Armus also interacts with LC3 family proteins and mediates fusion of starvation-induced autophagosomes with lysosomes (Popovic et al., 2012; Carroll et al., 2013). TBC1D5 interacts with the retromer complex, a Rab7 effector, and regulates retromer’s associations with membranes (Seaman et al., 2009). TBC1D5 also interacts with LC3 proteins, though it regulates autophagosome nucleation and expansion, i.e., earlier stages of autophagy than those regulated by Armus (Popovic et al., 2012; Roy et al., 2017). TBC1D15 also regulates autophagosome expansion, but in the context of mitophagy, rather than starvation-induced autophagy (Yamano et al., 2014). Like Armus and TBC1D5, TBC1D15 binds LC3 family proteins (Behrends et al., 2010; Yamano et al., 2014). TBC1D15 also binds the outer mitochondrial membrane protein Fis1, which may explain its ability to promote assembly of autophagosomes around damaged mitochondria (Onoue et al., 2013). In addition, TBC1D15 regulates dynamic lysosome-mitochondrion contacts that mark the sites of mitochondrial fission (Wong et al., 2018). Finally, Gyp7p is the GAP for the yeast Rab7 homolog Ypt7p (Vollmer et al., 1999; Brett et al., 2008). Little is known about Gyp7p, perhaps because phenotypes for its deletion or overexpression are fairly weak (Brett et al., 2008). No interactors for Gyp7p, other than Ypt7p, have been identified, nor does Gyp7p have any known role in autophagy.

## Bacterial Effectors/Virulence Factors That Modulate Rab7 Nucleotide Binding

Bacterial pathogens manipulate host intracellular trafficking in order to establish comfortable intracellular niches (Weber and Faris, 2018). This is accomplished by secreted bacterial proteins termed virulence factors or effectors (Alix et al., 2011). These effectors allow bacterial cells, engulfed via phagocytosis, to replicate inside host cells. Rab GTPases, including Rab7, are common targets of bacterial effector proteins (Stein et al., 2012). Two bacterial effectors manipulate the nucleotide-binding state of Rab7: RidL from *Legionella pneumophila*, and SopD2 from *Salmonella enterica* (serovar Typhimurium).

**FIGURE 7:**
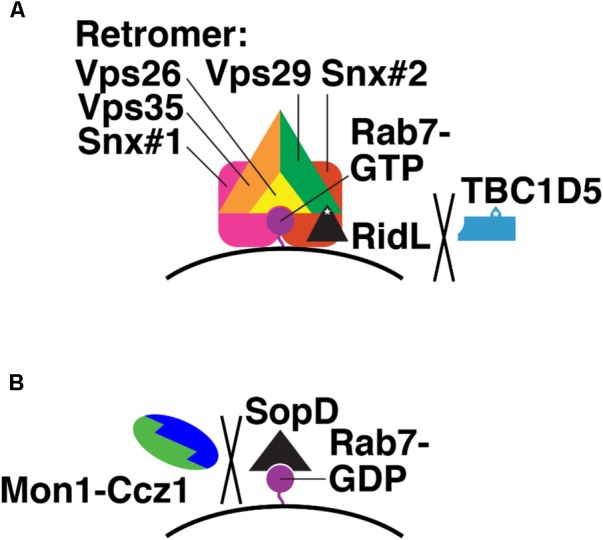
Bacterial effectors that modulate Rab7 nucleotide binding. **(A)** Mechanism of *Legionella* RidL: displacement of TBC1D5 from the Retromer complex and away from Rab7-GTR. Purple, Rab7-GTP; black, RidL; blue, TBC1D5; orange, Vps35; green, Vps29; yellow, Vps26; pink and red, sorting nexins. White asterisks denote binding sites in RidL and TBC1D5 for Vps29. **(B)** Mechanism of *Salmonella* SopD: binding Rab7-GDP and preventing Monl-Cczl GEF action. Purple, Rab7-GDP; black, SopD; green, Monl; blue, Cczl.

RidL is secreted by *L. pneumophila*, which causes Legionnaires’ Disease (Finsel et al., 2013). RidL is a substrate for the *Legionella* type IV secretion system (T4SS), which secretes bacterial proteins into host cell cytosol, across the bacterial inner and outer membranes and the limiting membrane of the *Legionella*-containing vacuole (LCV) that contains intracellular *Legionella* (Backert and Meyer, 2006). After secretion, RidL localizes to endosomes, where it binds the retromer via Vps29 (**Figure 7A**; Finsel et al., 2013). This interaction competitively blocks TBC1D5-Vps29 binding and displaces TBC1D5 into the cytosol (Barlocher et al., 2017; Yao et al., 2018). RidL does not displace Rab7 from LCVs; thus, Rab7 remains GTP-bound in the presence of RidL (**Figure 7A**; Barlocher et al., 2017). How RidL promotes *Legionella* survival and replication remains unclear. Perhaps the LCV acts as a sink for Rab7-GTP, reducing Rab7 levels on late endosomes and blocking transport of lysosomal hydrolases from late endosomes to LCVs.

The *S. enterica* (serovar Typhimurium) effector SopD2 directly blocks Rab7 nucleotide exchange (D’Costa et al., 2015). SopD2 is secreted via the syringe-like type III secretion system (T3SS) (Raffatellu et al., 2005), which pierces the host cell membrane surrounding the *Salmonella*-containing vacuole (SCV) where intracellular Salmonella reside (Moest and Meresse, 2013). SopD2 then blocks endocytic trafficking to lysosomes (D’Costa et al., 2015). SopD2 does this by preventing association of certain Rab7 effectors, in particular RILP and FYCO, which govern endosome, lysosome, and autophagosome mobility (Cantalupo et al., 2001; Jordens et al., 2001; Johansson et al., 2007; Pankiv et al., 2010; Olsvik et al., 2015), from the SCV (D’Costa et al., 2015). The precise molecular mechanism of SopD2 action has not yet been determined. However, purified SopD2 slows nucleotide exchange on purified Rab7, i.e., with no GEF present (D’Costa et al., 2015). This suggests that SopD2 binds and stabilizes Rab7-GDP (**Figure 7B**). This, in turn, would prevent Mon1-Ccz1 from catalyzing nucleotide exchange on Rab7-GDP and promoting delivery of hydrolytic enzymes to the SCV.

### Summary

Two known bacterial effectors modulate the nucleotide-binding state of Rab7. *Legionella* RidL displaces TBC1D5 from the retromer and thus probably locks Rab7 in its GTP-bound form. *Salmonella* SopD binds directly to GDP-bound Rab7 and blocks nucleotide exchange, i.e., activation. It is likely that more examples of the manipulation of Rab7 nucleotide binding by bacterial effectors will be found. Individual *Legionella* strains each secrete an estimated 300 proteins via the T4SS (Schroeder, 2017). Between the many *Legionella* strains that have been isolated, roughly 600 distinct T4SS substrates have been identified, and of these only 7 are conserved among all strains (Burstein et al., 2016). The repertoire of the Salmonella T3SS is more limited, with just 30 known substrates, but only about half of these have known functions (Figueira and Holden, 2012).

## Conclusion and Outlook

Rab7 and Ypt7p govern both endolysosomal trafficking and autophagy (Guerra and Bucci, 2016). They accomplish this by binding to a large and diverse set of effectors (Seals et al., 2000; Wurmser et al., 2000; Cantalupo et al., 2001; Jordens et al., 2001; Lazar et al., 2002; Pankiv et al., 2010; Sun et al., 2010). But before this can happen, the nucleotide-binding state of Rab7 and Ypt7p must be controlled in both time and space by the GEF and GAPs discussed here. Much work has gone into discovering these factors and elucidating their protein-protein and protein-lipid interactions (**Figures 4, 6 and Table 1**). These studies have made it possible to now seek a deeper level of understanding: How do Rab7/Ypt7p’s GEF and GAPs specify Rab7/Ypt7p activation at particular places and times?

Specific questions include, but are not limited to: Since Rab5 has many effectors besides Mon1-Ccz1 (Wandinger-Ness and Zerial, 2014), how is Mon1-Ccz1 recruitment by Rab5 triggered (**Figure 4**; Kinchen and Ravichandran, 2010)? What determines when Mon1-Ccz1 is deactivated by phosphorylation (Lawrence et al., 2014) and PI(3)P hydrolysis (Velichkova et al., 2010; Wu et al., 2014)? What is the functional significance of the binding of Rab7 GAPs to different LC3 proteins (**Table 1**)? What regulates LRRK1 phosphorylation of Armus/TBC1D2 (Toyofuku et al., 2015), and what triggers dephosphorylation? Does cargo binding to the retromer affect TBC1D5-retromer binding (Seaman et al., 2009), and vice versa? What mediates the switch between retromer and LC3 binding by TBC1D5 during autophagy (Roy et al., 2017)? Given that Armus and TBC1D5 both bind LC3 proteins (Popovic et al., 2012; Carroll et al., 2013; Roy et al., 2017), why does Armus mediate autophagosome-lysosome fusion (Carroll et al., 2013) and TBC1D5 regulate autophagosome formation (Popovic et al., 2012)? How does TBC1D15 shift from regulating mitochondrion-lysosome contacts (Wong et al., 2018) to regulating autophagosome growth around mitochondria (Yamano et al., 2014)?

Answers to these questions may come from an improved understanding of how the intermolecular interactions of Rab GEFs and GAPs change in time and space, and how they are affected by cellular metabolic state and the physical and chemical growth environment. Methods that could prove useful include FRET-based and split-enzyme/split-fluorescent protein biosensors for protein-protein interactions (Kerppola, 2006), proximity ligation and proteomics for unbiased sampling of protein-protein binding (Lobingier et al., 2017), and ion mobility mass spectrometry for isolating and characterizing protein-lipid complexes from native environments (Liu et al., 2017). Such approaches will allow development of quantitative, integrated models that explain how Rab7 nucleotide binding is regulated and, in turn, regulates endolysosomal trafficking and autophagy.

## Author Contributions

CS wrote the manuscript and prepared the figures.

## Conflict of Interest Statement

The author declares that the research was conducted in the absence of any commercial or financial relationships that could be construed as a potential conflict of interest.
